# Revealing the Enigma: A Deep Dive Into Submandibular Adenoid Cystic Carcinoma and Its Management

**DOI:** 10.7759/cureus.74660

**Published:** 2024-11-28

**Authors:** Oussama Slimani, Mohamed Moukhlissi, Soumiya Samba, Souad Margoum, Ahmed BenSghier, Soufiane Berhili, Loubna Mezouar

**Affiliations:** 1 Radiation Oncology, Faculty of Medicine and Pharmacy - Mohammed First University, Oujda, MAR; 2 Radiation Oncology, Faculty of Medicine and Pharmacy - Mohammed First University, oujda, MAR

**Keywords:** adenoid cystic carcinoma (acc), head and neck cancer, perineural invasion (pni), radiation, salivary glands carcinoma

## Abstract

Adenoid cystic carcinoma (ACC) of the submandibular gland is a rare and highly aggressive malignancy, distinguished by its tendency for perineural invasion and distant metastasis, particularly to the lungs and bones. The management of ACC is challenging due to its biological variability and the absence of specific randomized controlled trials to guide treatment. This report aims to encapsulate the clinical features, histological profile, diagnostic workup, and management options for submandibular gland ACC, with an emphasis on recent advancements in understanding and treatment. We present the case of a 42-year-old woman who presented with a progressively enlarging mass in the left submandibular region, subsequently diagnosed as ACC post-surgery. The patient received adjuvant radiotherapy following a multidisciplinary team decision, highlighting the importance of a tailored, multi-modal approach in the management of submandibular gland ACC. Ongoing research and collaborative efforts are essential for developing effective, personalized treatment strategies for this rare malignancy.

## Introduction

Submandibular adenoid cystic carcinoma (ACC) represents a rare but significant challenge in the realm of salivary gland malignancies. Despite its relatively small representation among head and neck cancers, the distinct histopathological characteristics and aggressive nature of ACC demand precise diagnostic and therapeutic approaches [1]. The carcinoma’s propensity for perineural invasion and distant metastases, primarily to the lungs and bones, contributes to its poor prognosis [2]. The management of submandibular gland ACC remains controversial, as the disease’s rarity and biological variability complicate the development of standardized treatment protocols.

Here, we present a case of a 42-year-old woman with a left submandibular mass, diagnosed as ACC post-surgery, illustrating the challenges and considerations in treating this condition. Despite achieving clear surgical margins, the patient underwent adjuvant radiotherapy due to the high risk of recurrence. The patient responded well to the treatment and remains under close follow-up, highlighting the importance of a multidisciplinary approach and the potential of targeted therapies in the management of submandibular gland ACC. This article aims to provide a comprehensive review of the clinical features, histological profiles, diagnostic workup, and management options for ACC of the submandibular gland, with particular emphasis on recent advancements in understanding and treatment strategies for this complex disease.

## Case presentation

**Figure 1:**
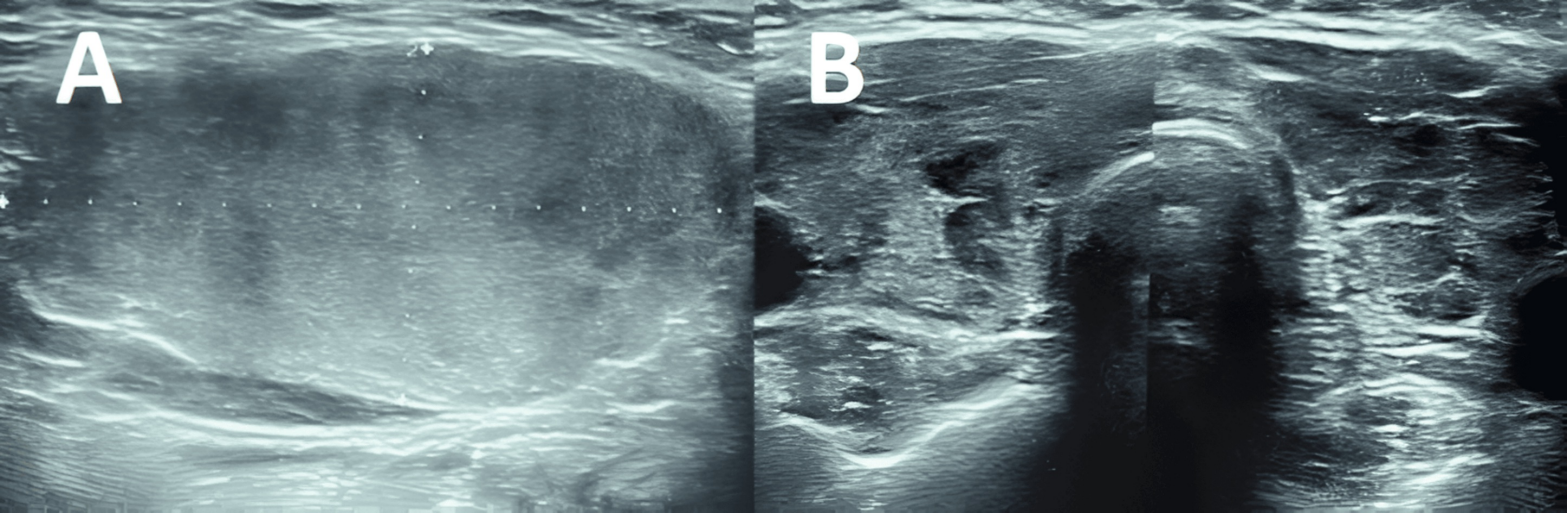
Ultrasound images showing a solid, non-homogeneous mass in the left submandibular gland, measuring 49.1 x 23 mm (A), and a goiter with diffuse hypoechoic areas without a clearly defined nodular image (B)

We present the case of a 42-year-old female with an unremarkable medical history who noticed a progressively enlarging left lateral cervical swelling over six months. Seeking medical attention, she underwent a standard biological workup and ultrasound neck. The ultrasound revealed a solid, nonhomogeneous mass in the left submandibular gland, measuring 49.1 x 23 mm, and a goiter with diffuse hypoechoic areas, lacking well-defined nodules, classified as Thyroid Imaging Reporting & Data System (TI-RADS™) 4A (Figure 1).

**Figure 2:**
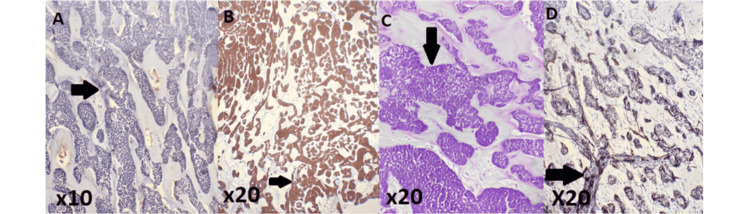
Histological and immunohistochemical findings pointing to the diagnosis of adenoid cystic carcinoma A: Negative staining of tumor cells with the anti-P40 antibody. B: Intense and diffuse staining of the tumor cells with the anti-CK7 antibody. C: The histological image shows a cribriform pattern typical of adenoid cystic carcinoma (ACC). The tumor features small, uniform basaloid cells in nests within a hyaline stroma. It includes pseudocystic spaces filled with basophilic mucin or hyaline material. The cells have small, hyperchromatic nuclei and scant cytoplasm while the fibrous stroma is dense and sclerotic, separating the nests. This pattern strongly suggests ACC, especially in salivary gland pathology. D: Positive staining of myoepithelial cells with the anti-AML antibody.

**Figure 3:**
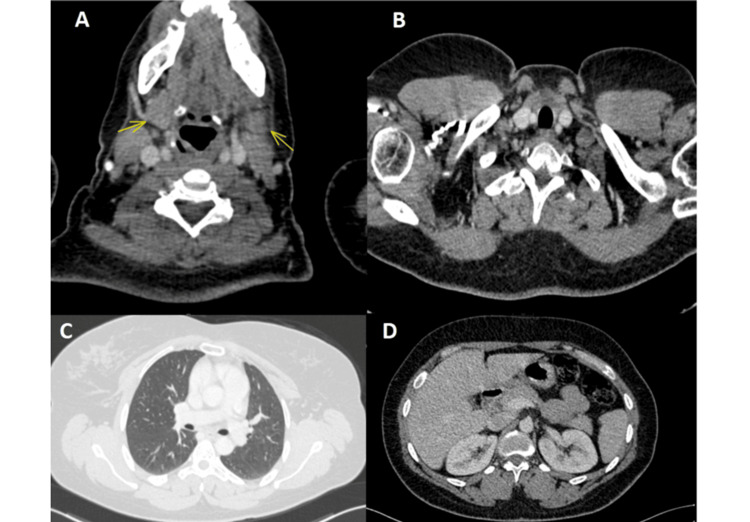
Postoperative cervico-thoraco-abdomino-pelvic CT scan A and B: Axial sections of the cervical region. A shows asymmetry in the size of the submandibular glands related to the surgical procedure. B reveals evidence of thyroidectomy (empty thyroid bed). C: Axial section of the thoracic region, parenchymal window, showing no secondary pulmonary lesions. D: Axial section of the abdominal region, with no secondary lesions detected in this region.

The patient subsequently underwent mass resection and total thyroidectomy. Histopathological examination revealed multinodular thyroid hyperplasia with lymphocytic thyroiditis and a salivary-type tumor, necessitating immunohistochemical analysis, which confirmed the diagnosis of adenoid cystic carcinoma with resection margins of less than 2 mm (Figure 2). A postoperative staging workup, consisting of a cervico-thoraco-abdomino-pelvic CT scan, revealed asymmetry in the size of the submandibular glands related to the surgical procedure, signs of thyroidectomy with an empty thyroid bed, no cervical lymphadenopathy, and no suspicious lesions indicative of malignancy in the explored regions (Figure 3).

**Figure 4:**
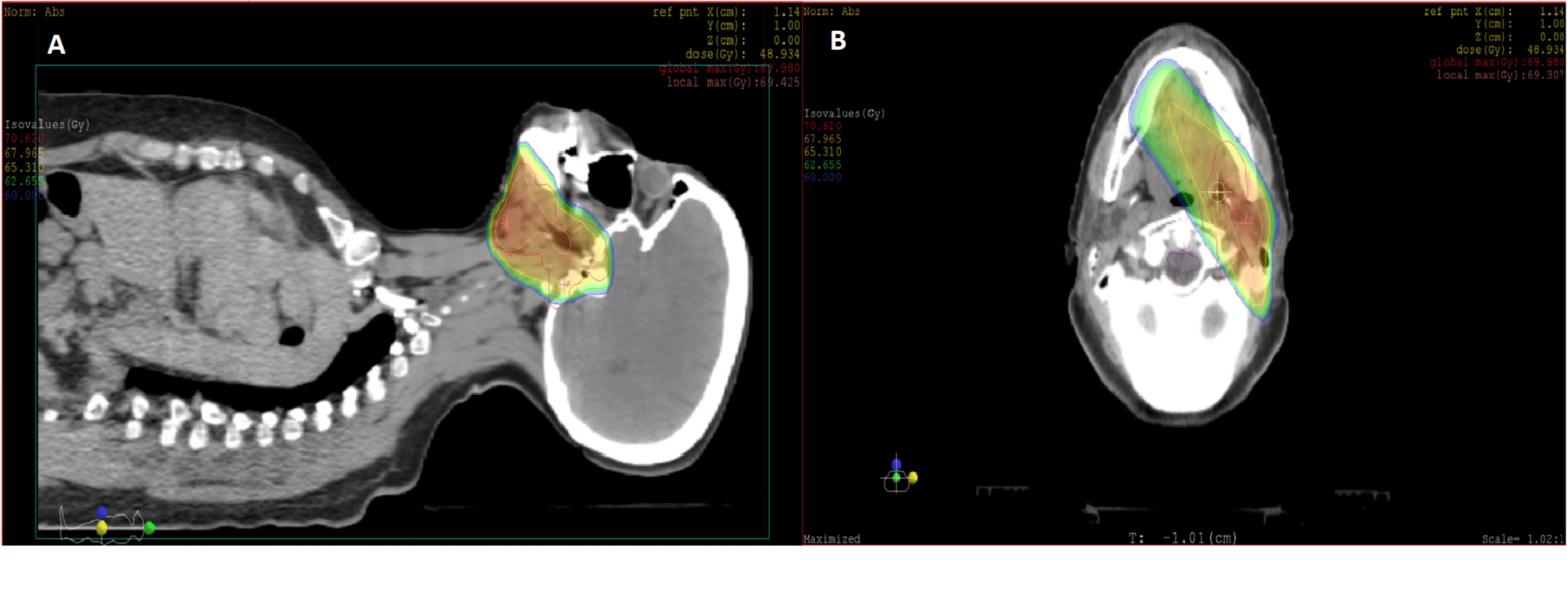
Dosimetric assessment of radiotherapy colorwash dose coverage of primary tumor bed A: Saggital section. B: Axial section.

This case was discussed in a multidisciplinary meeting, leading to the decision to administer adjuvant radiotherapy. The patient received three-dimensional conformal radiotherapy at the tumor bed and along the path of the affected cranial nerves, with a total dose of 66 Gy, fractionated in 2 Gy per session (Figure 4). The patient tolerated the treatment well, experiencing only grade I radiation dermatitis, which improved with symptomatic treatment. She is currently on thyroid hormone replacement therapy and is maintained on close, regular follow-up with clinical examinations and imaging.

## Discussion

ACC of the submandibular gland is a rare malignancy, presenting significant management challenges due to its aggressive growth pattern, characterized by perineural invasion and a propensity for distant metastases, primarily to the lungs and bones [2]. The management of ACC necessitates a multidisciplinary approach, involving surgery, radiation oncology, and often systemic therapy. The optimal treatment strategy remains elusive, hindered by the absence of large-scale randomized controlled trials specifically addressing ACC of the submandibular gland.

Accurate diagnosis is critical for the effective management and prognosis of ACC. Histopathologically, ACC displays a distinctive biphasic pattern comprising cribriform, tubular, or solid growth patterns within a mucinous and/or hyaline stroma [3]. Immunohistochemical staining, utilizing markers such as CK7, CK14, and S100 aids in differentiating ACC from other salivary gland tumors. Furthermore, molecular studies have identified recurrent genetic alterations in ACC, such as chromosomal translocations involving the MYB and, less frequently, MYBL1 genes, which could serve as potential diagnostic markers or therapeutic targets [1].

ACC typically presents as an asymptomatic, slow-growing mass, with symptoms such as mild facial asymmetry and dysphagia often overshadowed by more common conditions like fever or cough [4]. Despite its indolent clinical course, ACC is marked by a slow and insidious local invasion pattern, frequently spreading perineurally along branches of the trigeminal nerve, leading patients to report facial numbness or pain [5]. The clinical trajectory of ACC varies widely, with some patients exhibiting indolent behavior and others developing high-grade, widely metastatic disease. Consequently, individualized management strategies based on specific risk profiles are essential.

Standard management of submandibular gland ACC involves surgical resection with negative margins [6,7]. However, the gland's proximity to vital anatomical structures and frequent perineural involvement often complicate complete resection. Adjuvant radiation therapy is commonly employed to mitigate local recurrence rates, especially in cases with positive margins or high-risk features like perineural invasion [8,9]. Given the high risk of local recurrence in ACC, postoperative radiotherapy is generally recommended. Indications for postoperative radiotherapy include incomplete surgical resection, positive or close margins, and the presence of perineural invasion (PNI) [8,10]. According to the recommendations of the NCCN (National Comprehensive Cancer Network), adjuvant radiotherapy is often indicated for cystic adenoid tumors of the salivary glands, especially in cases of locally advanced tumors or positive or insufficient surgical margins. Retrospective studies have demonstrated locoregional control rates ranging from 36% to 93% for unresectable or incompletely resected salivary gland tumors, including ACC [9,11]. The impact of postoperative radiotherapy on overall survival is debated; while some studies report a survival advantage with surgery alone, others find no significant survival benefit despite improved locoregional control [12,13].

In cases where adjuvant radiation therapy is necessary, the primary tumor bed must be included in the treatment plan. Elective treatment of cranial nerve pathways, while beneficial, presents challenges due to the increased toxicity associated with extensive treatment volumes. Balancing the anticipated benefits of covering elective cranial nerve pathways against the risks of expanding treatment volumes is crucial. Given ACC’s propensity for perineural tumor spread, considering the elective safeguarding of at-risk cranial nerves at their primary tumor sites is warranted [14]. The lymphatic system is seldom involved in ACC, making routine neck therapy unnecessary unless histological or imaging evidence suggests regional involvement. Advanced T-stage disease, however, may necessitate neck treatment due to a higher likelihood of nodal involvement [15].

When the submandibular gland is affected, a thorough examination of the lingual nerve (V3), chorda tympani (VII), and hypoglossal nerve (XII) is crucial. Malignancies may spread along the lingual nerve to the mandibular nerve at the skull base via the foramen ovale, with potential involvement of the chorda tympani. The close proximity of the hypoglossal nerve to the submandibular gland also raises the possibility of its involvement (Table 1) [16].

**Table 1:** Cranial nerves at risk based on ACC primary site Source: [16] ACC: adenoid cystic carcinoma

Primary ACC Tumor Site	Cranial Nerves at Risk	Origin at Base of Skull	Additional Cranial Nerves at Risk via Inter-Nerve Connections
Submandibular Gland	V3, XII (deep lobe involvement)	Foramen ovale Hypoglossal canal	VII, via chorda tympani (rarely included in elective volumes as involvement is rare)
Parotid Gland	VII	Stylomastoid foramen	V3, via auriculotemporal nerve
Hard Palate	V2	V2:Foramen rotundum	VII, via greater superficial petrosal nerve and vidian nerve

Radiation doses for ACC are determined by balancing the risks of treatment toxicity against the potential disease burden and the risk of skull base failure. Although specific data on appropriate dosing for ACC is limited, general radiation therapy principles suggest a 60 Gy dose for the primary tumor bed in postoperative treatment, increasing to 64 Gy for close and 66 Gy for positive microscopic margins. Elective neural coverage should receive 50-60 Gy, with lower doses preferred for the skull base. If significant perineural spread is present, doses to affected cranial nerve pathways may be increased to 66 Gy. Standard neck treatment algorithms apply in cases of nodal involvement. For unresectable ACC, definitive radiation therapy doses of 70 Gy are administered to the gross tumor and involved nerves, with careful dose reduction beyond these areas. These doses are typically delivered in 2 Gy fractions [17].

Classical toxicities associated with head and neck irradiation are well-documented. Involvement of the skull base and cranial nerve pathways increases the risk of severe morbidity, including permanent blindness, hearing loss, temporal lobe necrosis, and cranial neuropathies. The risk of radiation-induced cranial neuropathies may increase over time due to their long latency. Despite extensive treatment efforts, ACC of the submandibular gland is characterized by a high recurrence rate and distant metastasis, underscoring the need for novel therapeutic strategies. Ongoing research into the molecular progression of ACC is expected to identify new therapeutic targets, potentially improving outcomes for this challenging condition. Additionally, collaborative multicenter studies are necessary to develop consensus guidelines for ACC management that integrate advancements in diagnostic modalities, surgical techniques, and targeted therapies [18].

## Conclusions

Submandibular adenoid cystic carcinoma presents a significant clinical challenge due to its aggressive nature, high recurrence rate, and potential for distant metastasis. Despite advancements in diagnostic techniques and therapeutic strategies, the management of ACC remains complex, necessitating a multidisciplinary approach. This case underscores the critical role of accurate diagnosis, surgical resection with negative margins, and adjuvant radiotherapy in improving patient outcomes. However, the rarity of the disease and lack of comprehensive clinical trials underscore the need for ongoing research to refine treatment protocols and identify novel therapeutic targets. The development of targeted therapies and the integration of molecular diagnostics into clinical practice hold promise for enhancing the management and prognosis of patients with ACC. Continued efforts in collaborative research and long-term clinical follow-up are imperative to achieve optimal care for individuals affected by this challenging malignancy.
